# Design and Comparative Numerical Analysis of AlSi10Mg PBF-LB/M Manufactured TPMS Lattice Structures for Improved Mechanical Performance

**DOI:** 10.3390/ma18214934

**Published:** 2025-10-28

**Authors:** Laura Luran Sun, Nikola Milenkovski, Kartikay Awasthi, Xuan Bach Nghiem, Nicola Mongelli, Eckhard Kirchner, Christian Mittelstedt

**Affiliations:** 1 Product Development and Machine Elements, Technical University of Darmstadt, 64287 Darmstadt, Germany; 2 Lightweight Engineering and Structural Mechanics, Technical University of Darmstadt, 64287 Darmstadt, Germany

**Keywords:** additive manufacturing, triply periodic minimal surface, AlSi10Mg, numerical simulation, laser powder bed fusion, lattice structures

## Abstract

Additive manufacturing enables the fabrication of lightweight structures with complex geometries, offering significant potential in aerospace and biomedical applications. Triply periodic minimal surface (TPMS) lattice structures are of particular interest due to their geometry. However, their intricate geometries pose challenges for both experimental characterization and numerical simulation. This study numerically investigates the effective mechanical properties and dynamic response of AlSi10Mg TPMS structures produced by laser powder bed fusion (PBF-LB/M). Using a micro–mesoscale approach with periodic boundary conditions, Young’s modulus, shear modulus, and Poisson’s ratio in the elastic region using the Johnson–Cook plasticity model are analyzed. Finite element simulations of the representative volume element (RVE) are employed to assess energy absorption and damage evolution under high strain rates, incorporating a ductile damage model. The performance of sheet-based TPMS lattices, namely, Schwarz–Primitive, Gyroid, Schwarz–Diamond, and IWP, is compared with strut-based lattices, namely, BCC and FCC, across volume fractions of 20–40%. Results demonstrate the superior stiffness and energy absorption of TPMS lattices, where Schwarz–Diamond and IWP outperformed the other structures, highlighting their advantages over conventional strut-based designs. This comprehensive numerical framework provides new insights into the high strain-rate behavior of TPMS structures and supports their design for demanding engineering applications.

## 1. Introduction

Triply periodic minimal surface (TPMS) structures have gained significant attention due to their high strength-to-weight ratio, superior energy absorption characteristics, and tunable mechanical properties [1,2,3,4,5]. TPMS structures are mathematical models that describe surfaces with a mean curvature of zero, meaning they are neither concave nor convex overall [4,6]. This unique geometric property minimizes total surface energy and stress concentrations, enhancing structural stability and resistance to deformation [7]. Unlike conventional strut-based lattice structures, TPMS designs feature continuous curvatures that distribute stress more evenly, reducing stress localization at nodes and improving fatigue resistance [8]. As a result, TPMS structures exhibit superior mechanical strength and stiffness compared to strut-based lattices [9,10].

Due to the geometric complexity, conventional manufacturing methods are unsuitable for the fabrication of TPMS structures. Additive manufacturing technology, such as laser powder bed fusion (PBF-LB/M), allows for the fabrication of these intricate designs. Given the resource-intensive and time-consuming nature of PBF-LB/M-manufactured TPMS structures and its post-processing requirements, it is not feasible to conduct large numbers of experimental trials [11]. Therefore, accurate and efficient numerical simulations are essential to reduce experimental effort while still capturing the key material and structural behaviors [12]. Numerical modeling in the form of finite element simulation offers an alternative for exploring mechanical behavior, but it faces challenges such as complex meshing requirements and high computational costs [13,14,15]. The accurate material characterization of additively manufactured metals is challenging because their properties are strongly influenced by the chosen process parameters as well as inevitable process fluctuations [16,17]. Furthermore, the lack of robust damage and failure models for PBF-LB/M-fabricated TPMS structures limits predictive accuracy.

Previous studies on metal-based TPMS structures, both experimental [18,19,20] and numerical [15,21], have largely focused on effective elastic properties as well as low strain-rate conditions. Studies have also compared the effectiveness of different element types in simulating mechanical properties. Shell elements generally show increasing divergence from the results obtained using solid elements as the wall thickness, and therefore, volume fraction increases, where the solid model matches the experimental results more accurately [15,22,23]. Under high-strain loading conditions, especially with high-volume fractions, this discrepancy further increases, as shell elements have limited ability to capture local phenomena such as material flow. In contrast, solid elements show greater accuracy in representing complex behaviors such as post-yield deformation [15].

Comparative investigations across different TPMS topologies remain limited, and studies addressing their strength-to-weight relationship, damage mechanisms and high strain-rate responses are particularly scarce. Many comprehensive numerical studies cover polymers instead of metal-based materials [24,25]. To address these gaps, the present study numerically investigates the mechanical performance of various AlSi10Mg TPMS structures, alongside conventional strut-based lattices for comparison, under dynamic uniaxial compression. Finite element simulations are used to evaluate the effective elastic properties Young’s modulus, shear modulus, and Poisson’s ratio, as well as energy absorption characteristics and damage evolution at high strain-rates of 37.5 1/s. By establishing the relationship between topology, volume fraction, and the resulting mechanical performance, this study provides insights into the deformation mechanisms that influence the high strength-to-weight ratio of TPMS structures.

## 2. Materials and Methods

### 2.1. Homogenization

The Hill–Mandel condition must be satisfied for the transition from macro to micro scales. If the repeated unit cell (RUC) repeats periodically, and the surrounding macroscopic continuum averages out geometrically over its volume, then the mechanical work at any point in the continuum equals the volume-averaged virtual work of a representative volume element (RVE) of the structure. Additionally, the RVE must be large enough to capture the essential features of the whole lattice structure, including the distribution of material and mechanical interactions, and typically be composed of periodic RUCs [26,27]. The homogenized elastic strain ε, and Cauchy stress tensor σ, can be determined through the following conditions [26]:

(1) $$\sigma :\varepsilon =\langle \sigma^{m}:\varepsilon^{m}\rangle ,$$

where the subscript m denotes the micro–mesoscopic quantities. For any microscopic continuum, the averages over the initial configuration of the RUC are given as follows:

(2) $$\langle \left(\cdot \right)\rangle =\frac{1}{V}\int_{V}\left(\cdot \right)dV,$$

where *V* is the volume of the RUC. The effective deformation gradient $\langle F\rangle$ is defined as follows:

(3) $$F=\frac{\partial x}{\partial X},\;\;\;F=\langle F^{m}\rangle .$$

Here x and X define the position of the material point in the deformed and initial configuration, respectively.

### 2.2. Elastoplasticity and Damage Model

Material characterization can be widely divided into three segments: the elastic region, the plastic region, and the softening region leading to fracture. First of all, the strain can be additively decomposed as follows:

(4) $$\varepsilon =\varepsilon_{e}+\varepsilon_{p}$$

where $\varepsilon_{e}$ and $\varepsilon_{p}$ are the elastic and plastic strains, respectively. The material is assumed to be isotropic. Plasticity is defined using the Johnson–Cook plasticity model. Isothermal loading conditions are assumed, and temperature-dependent terms are ignored. The equivalent von Mises stress $\sigma_{eq}$ obtained during plastic deformation using the Johnson–Cook model [28] is defined in terms of the deviatoric stress tensor s as follows:

(5) $$\sigma_{eq}=\sqrt{\frac{3}{2}s:s}=\left[A+B\varepsilon^{n}\right]\left[1+C\ln{\dot{\varepsilon }}^{*}\right],$$

where *A* = yield stress, *B* = tangent modulus, *n* = hardening exponent, *C* = strain-rate hardening factor, and ${\dot{\varepsilon }}^{*}=\frac{\dot{\varepsilon }}{{\dot{\varepsilon }}_{0}}$ where $\dot{\varepsilon }=$ true strain rate, and ${\dot{\varepsilon }}_{0}=$ reference strain rate at which Johnson–Cook plasticity properties were measured.

Damage initiation is characterized by a phenomenological model for ductile failure (Kolmogorov integral criterion) [29], where the damage initiation is governed by a state variable $\omega_{D}$ such that

(6) $$\omega_{D}=\int_{0}^{\varepsilon_{f}}\frac{d\varepsilon_{p}}{\varepsilon_{f}(\eta )},$$

where $\varepsilon_{f}$ is the plastic strain at damage initiation, which is a function of stress triaxiality η, and $d\varepsilon_{p}$ is the plastic strain accumulated during deformation. Damage is initiated when $\omega_{D}=1$. Following the initiation of damage, the material will undergo softening before complete failure. In this study, material softening was modeled using the J-Integral form of fracture toughness, also known as fracture energy. Fracture energy ($G_{c}$) is calculated based on the stress–intensity factor $K_{IC}$ [30]:

(7) $$G_{c}=\frac{K_{IC}^{2}}{E},$$

where *E* is the Young’s modulus of the material. It should be noted that this standard relationship implicitly assumes plane–strain conditions. This represents a simplification, as the thin-walled sheet structures investigated in this study may be more accurately represented by a plane–stress state. The constants for elasticity and Johnson–Cook plasticity were obtained from an experimental study on PBF-LB/M AlSi10Mg specimens in Table 1 [31]:

Further, the damage initiation and damage evolution were referred from the EOS material datasheet and literature on experimental testing of fracture energy in Table 2, both for PBF-LB/M AlSi10Mg [30,32].

For this study, the general ductile damage model was simplified to use a constant damage initiation threshold governed by a single stress triaxiality and its corresponding plastic strain at damage initiation. A full experimental characterization of this relationship is currently not available in the literature. In this numerical framework, the Johnson–Cook plasticity model and the damage evolution model govern separate aspects of the material response. This implementation assumes that the fracture energy $G_{c}$ itself is rate independent. This decouples the two phenomena by assigning rate effects solely to hardening in the plastic region and damage evolution solely to post-initiation softening.

### 2.3. Energy Absorption

**Figure 1:**
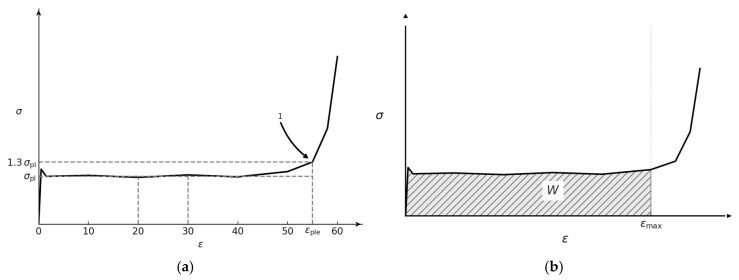
Quantification of energy absorption as per ISO 17340:2020 (**a**) $\varepsilon_{max}$ at 1.3× plateau stress; (**b**) energy absorption as area under the stress–strain curve [34].

Energy absorption is the ability of a body or material to transform kinetic energy into other forms of energy through elastic and plastic deformation, mechanical instabilities, and failure [33]. Energy absorption is directly linked to the stress–strain response of a body as shown in Figure 1a. The ISO standard 17340:2020 [34] details the methodology and guidelines for ductile metal testing in porous or cellular media. The standard defines cellular material as having a porosity of 50% or more, entailing compression between strain rates of $1\;s^{-1}$ to $1{0^{3}\;s}^{-1}$. Energy absorption can be quantified in two forms: volumetric (*W*) and specific energy absorption (*SEA*), mathematically represented as follows:

(8) $$W=\int_{0}^{\varepsilon_{u}}\sigma (\varepsilon )d\varepsilon ,\;\;\;\;SEA=\frac{W}{m}$$

where σ(ε) is the stress at strain ε, $\varepsilon_{u}$ is the strain limit and m is the mass of the body. It may also be interpreted as the area under the stress–strain curve up to the strain limit as shown in Figure 1b. The ISO standard defines this strain limit as 50% or 1.3× the plateau strain. For the current study, the 50% strain limit and a strain rate of 37.5 s^−1^ is considered.

### 2.4. Implicit Modeling

TPMS lattices are distinguished by their zero mean curvature $H=\frac{k_{1}+k_{2}}{2}=0$, with $k_{1}$ and $k_{2}$ being the principal curvatures, giving them mathematically minimal, self-supporting surfaces that repeat regularly throughout the three-dimensional space. The study implements TPMS geometries implicitly with a truncated Fourier level-set function as follows [35]:

(9) $$\phi \left(r\right)=\sum_{k=1}^{K}A_{k}\cos\left(\frac{2\pi \left(h_{k}\cdot r\right)}{\lambda_{k}}+p_{k}\right)=c,$$

where $h_{k}=$ reciprocal lattice vectors, r= spatial position vector in $R^{3}$, $A_{k}=$ amplitude factor, $\lambda_{k}=$ wavelength, $p_{k}=$ phase shift, and c= constant that controls the relative density of the RVE.

**Figure 2:**
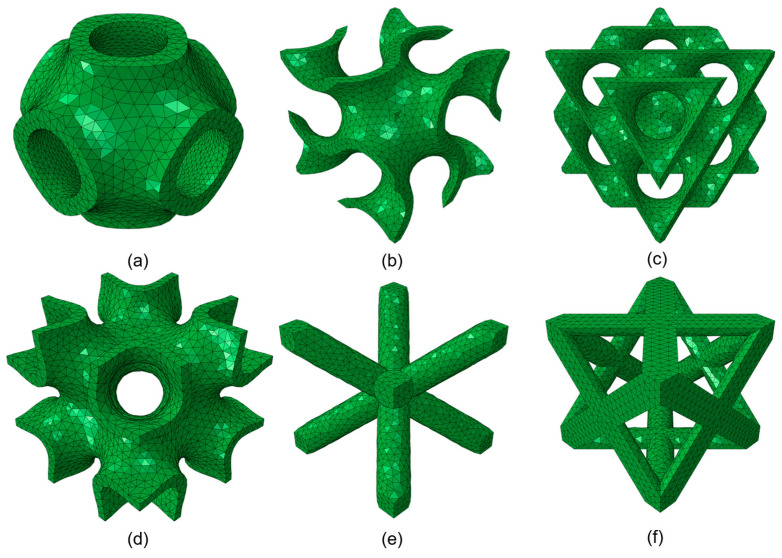
Repeating unit cells (RUCs) of lattice structures. (**a**) Primitive; (**b**) gyroid; (**c**) diamond; (**d**) IWP; (**e**) BCC; (**f**) FCC.

Level-set equations of the form $f\left(x,y,z\right)=\phi \left(x,y,z\right)-c=0$ can be derived through this representation, allowing for the precise creation of TPMS geometries. Regions where $f\left(x,y,z\right)<0$ are solid, while areas with $f\left(x,y,z\right)>0$ are void. In this study, the mechanical performance of the following TPMS structures is investigated [20]:

(10) $$\mathrm{Primitive}:\;\;cos\left(Ax\right)+cos\left(Ay\right)+cos\left(Az\right)=c$$

(11) $$\mathrm{Gyroid}:\;\;sin\left(Ax\right)cos\left(Ay\right)+sin\left(Ay\right)cos\left(Az\right)+sin\left(Az\right)cos\left(Ax\right)=c$$

(12) $$\begin{array}{c} \mathrm{IWP}:\;\;2\left(cos\left(Ax\right)cos\left(Ay\right)+cos\left(Ay\right)cos\left(Az\right)+cos\left(Az\right)cos\left(Ax\right)\right)-\\ \left(cos\left(2Ax\right)+cos\left(2Ay\right)+cos\left(2Az\right)\right)=c \end{array}$$

(13) $$\begin{array}{c} \mathrm{Diamond}:\;\;sin\left(Ax\right)sin\left(Ay\right)sin\left(Az\right)+sin\left(Ax\right)cos\left(Ay\right)cos\left(Az\right)+\\ cos\left(Ax\right)sin\left(Ay\right)cos\left(Az\right)+cos\left(Ax\right)cos\left(Ay\right)sin\left(Az\right)=c \end{array}$$

$$\mathrm{With}\;A=\frac{2\pi }{l}$$

where l is the side length of the RUC. Solid TPMS lattices can be realized in two ways. Skeletal structures are created by filling one of the two minimal-surface sub-volumes, so that density scales with the filled fraction [4]. In contrast, sheet structures, which are used in this study, can thicken the minimal surface. Target densities are achieved by varying the thickness, allowing for continuous surfaces even at very low relative densities, with the limiting factor being the resolution of the manufacturing system. Figure 2 shows the TPMS and strut-based structures investigated in this study.

### 2.5. Geometrical Properties and Design

The TPMS geometrical properties, such as RUC size, nominal thickness, and nominal relative density, were parameterized, respectively, and their raw stereolithography (STL) format was created using their implicit functions and parameters. The shell of the body-centered cubic RUC was generated by the CAD software Autodesk Fusion (https://www.autodesk.com/products/fusion-360/overview). The finite element software ABAQUS/CAE 2023 was used for the FE simulations. The STL models were imported into ABAQUS and converted to CAD using virtual topology to create the full FE model. The unstructured and pre-generated mesh was deleted, and the shells were remeshed accordingly using the values obtained from the mesh convergence study, which is discussed briefly in Section 3.1. The mesh was corrected on all boundary surfaces to achieve periodicity for periodic boundary conditions. Finally, the mesh elements were converted to C3D10 solid elements in quadratic order. The chosen unit cells have a bounding box dimension of 5 mm, with a relative density of 20%, 30%, and 40%.

Unlike a single unit cell, bulk lattice structures undergo a sequential collapse of unit cells, characterized by compressive failure. To simulate this phenomenon, an explicit solver was used, and the mesh elements were switched from C3D10 to its modified version, C3D10M, which is suitable for large deformation and capturing plastic behavior. Additionally, general contact was applied globally on the lattices and the rigid plates. Hard contact and friction penalty with a coefficient of 0.1 were defined to capture the layer collapses and the interaction between the lattice layers and the plates.

### 2.6. Boundary Conditions

**Figure 3:**
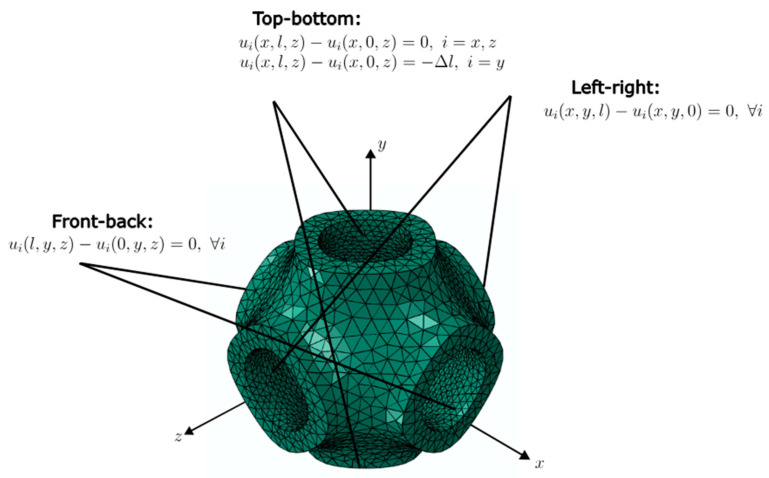
Boundary conditions for uniaxial compression.

**Figure 4:**
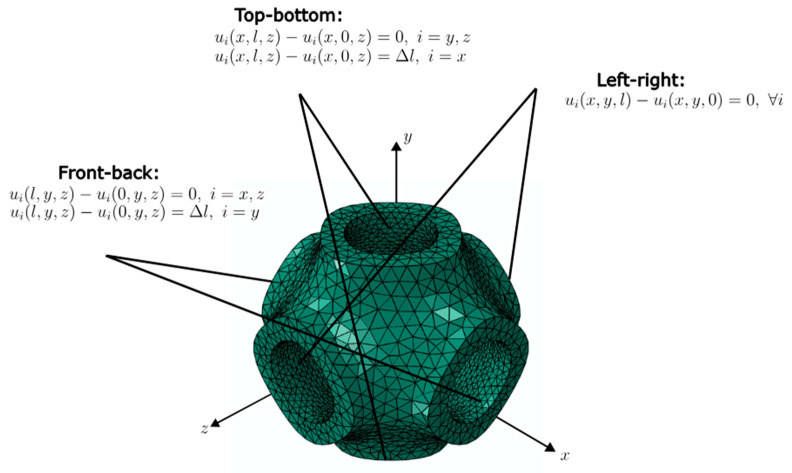
Boundary conditions for pure shear.

For the homogenization of the effective performance of the lattice structures, the simulations were carried out on the RUC. The unit cell was subjected to uniaxial compression and pure shear loading scenarios. The deformation of the homogenized unit cell is specified in terms of a displacement gradient H=F−I. Here, the Dirichlet boundary condition “d” and the periodic boundary condition “p” were applied on the boundary nodes:

(14) $$\begin{array}{c} d:\;u=Hx\\ p:\;u\left(x^{+}\right)-u\left(x^{-}\right)=H\left(x^{+}-x^{-}\right), \end{array}$$

where u is the displacement, and $x^{+}$, $x^{-}$ are the positions of the nodes on opposite boundaries, respectively. For the effective elastic modulus and shear modulus, the analyses were performed under strain-controlled loading, where the uniaxial compression and shear loads corresponding to 5% strain were applied via prescribed strain. Figure 3 and Figure 4 show the boundary conditions applied for the uniaxial compression and pure shear load cases, respectively.

**Figure 5:**
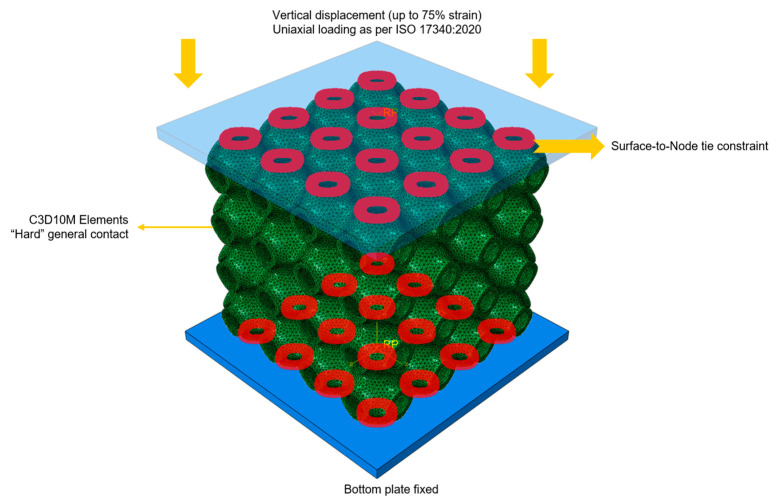
Boundary conditions for energy absorption on the RVE.

The energy absorption of bulk lattice structures was determined in a uniaxial compression test. The bottom side was fixed, while a displacement until 75% strain was applied to the top plate. A strain rate of 37.5 s^−1^ was chosen to simulate a maximum deceleration of 40 g on a standard impact attenuator prescribed in the FSAE regulations [36]. The resulting load–displacement data were collected to calculate stress–strain values. From these simulations, the absorbed energy was calculated as the area under the stress–strain curve. Figure 5 shows the applied boundary conditions on the RVE for energy absorption.

## 3. Results and Discussion

### 3.1. Mesh Convergence

**Figure 6:**
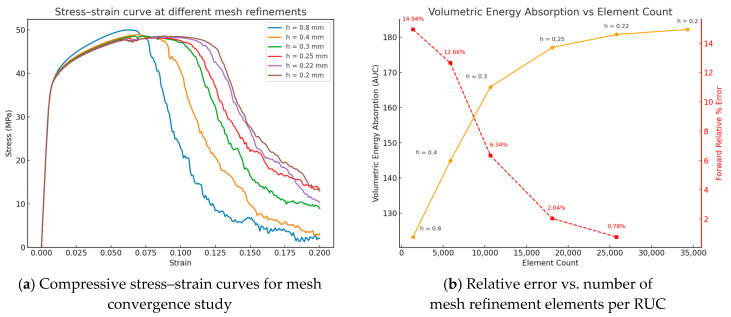
Exemplary mesh convergence study on the TPMS Diamond.

A mesh convergence study was performed to ensure the accuracy of the FEM using finite strain solid elements as depicted in Figure 6. Boundary conditions identical to energy absorption until a strain of 0.2 were applied to a Diamond TPMS unit cell with varying element count. The element count in the mesh was controlled by a mesh element size varying from 0.2 mm to 0.8 mm with mesh refinements being in a roughly even step of element count. The material response includes the elastoplastic behavior as well as the damage initiation and evolution model. The stress–strain curves show that the elastoplastic behavior agrees and converges quickly, with deviations in the material softening zone. The ideal element size was found using the forward relative percentage error. The volumetric energy absorption (represented by the area under the curve) is measured for all the runs. From the fourth refinement, the error already falls under 1%. This concludes that no further mesh refinement was required. Analogously, the corresponding number of elements for all other lattice unit cells in this study are found with the same element rate.

### 3.2. Normalized Effective Properties

**Figure 7:**
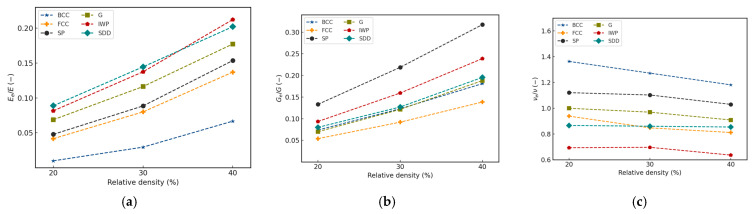
Normalized (**a**) Young’s modulus, (**b**) shear modulus, (**c**) Poisson’s ratio at 5% for relative densities of 20–40%.

The normalized Young’s modulus of the lattice unit cells is shown in Figure 7a. Overall, it is apparent that the Young’s modulus increases exponentially and proportionally to the relative density of the lattices. TPMS lattice types outperformed strut-based lattices in terms of resisting uniaxial strain, where BCC is the weakest across all values of density. The FCC roughly matches the weakest TPMS structure, which is the Primitive, in terms of Young’s modulus, while the gyroid consistently finds itself in the average. As the material becomes denser, IWP showed a rapid increase and surpassed the Diamond at a density of 40%, which shows the highest values among all other lattices at relative densities of 20% and 30%.

The normalized shear modulus is presented in Figure 7b. The Primitive’s homogenized shear modulus was the highest among all other lattices and all relative density levels, followed by IWP, while FCC’s performance against shearing was the lowest. Gyroid, Diamond, and BCC give the average shear modulus, which gradually increases proportionally to the change in relative density. A possible explanation for this is that the Schwarz Primitive lattice forms a network by connecting “channels” along the three principal axes. When a shear force is applied the surfaces oriented parallel to the load, it provides direct, continuous walls that strongly resist this sliding deformation. The force is transmitted primarily through in-plane shear and stretching of these surfaces resulting in the Schwarz Primitive lattice exhibiting a higher shear modulus.

Looking at the normalized Poisson’s ratio as shown in Figure 7c, the strut-based BCC lattice shows the highest values. For most lattice types, there is an anti-proportional relationship between relative density and normalized Poisson’s ratio. The IWP lattice shows the lowest values across all three relative densities, making it the most resistant towards lateral deformation under axial loads. This might be due to the IWP lattice’s inward-curved geometry, which is topologically constrained in a way that causes them to fold under uniaxial compressive loading. This inward-pulling mechanism due to the lattice’s natural tendency to fold, which counteracts the lateral expansion, resulting in a low effective Poisson’s ratio.

### 3.3. Representative Volume Fraction

In periodic cellular material, it is a known phenomenon that effective properties change diminishingly as more RUCs are added. Beyond a certain point, the added cost of computation does not correspond to a significant increase in accuracy of the measured effective (homogenized) properties. This volume is known as the representative volume element (RVE). Therefore, a series of simulations, with varying bulk lattice sizes from 1×1×1 to 5×5×5, under uniaxial compressive loading is performed. The stress–strain response for each is plotted, and the peak stress, as well as the Mean Percentage Error (MPE) of the stress–strain response, is measured.

**Figure 8:**
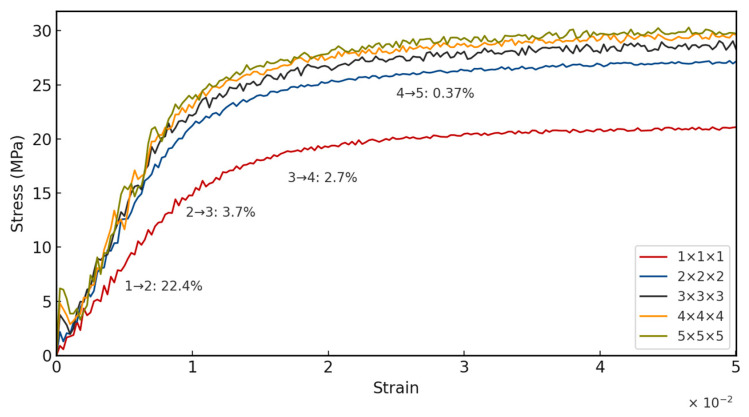
Representative volume element study.

As shown in Figure 8, it is observed that the change between 4×4×4 and 5×5×5 is already under the 1% threshold, while having roughly half the number of unit cells, and consequently half the number of elements. Therefore, for studying the energy absorption, a bulk lattice size of 4×4×4 is considered.

### 3.4. Energy Absorption

**Figure 9:**
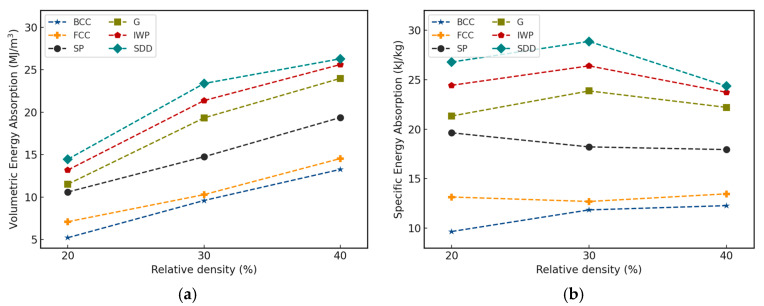
(**a**) Volumetric and (**b**) specific energy absorption characteristics of different lattices across all relative densities.

The stress–strain relation is obtained by studying the reaction force on the top plate of the compression assembly. To obtain the energy absorbed, the area under the curve is calculated using the trapezoidal rule. With 50% as the upper strain limit, the energy absorption is summarized in Figure 9.

Figure 9 clearly shows that TPMS lattices outperform strut-based lattices at the same relative density in terms of energy absorbed per volume or unit mass. Gyroid, IWP, and Schwarz Diamond show a significant increase in volumetric energy absorption with increasing relative density, with IWP and Schwarz Diamond performing similarly across all relative densities. As common applications are lightweight structures, the energy absorption is divided by the mass of the lattice structure to obtain the specific energy absorption as shown in Figure 9b. TPMS lattices exhibited the highest specific energy absorption at a relative density of around 30% with the exception of Schwarz Primitive, which performed best in terms of specific energy absorption at 20%. Overall, Schwarz Diamond showed the best potential for energy absorption across all other lattices, which is denoted by the localized elastic recovery. This is in alignment with experimental results from literature [18,19]. On the contrary, strut-based types like BCC and FCC went through a longer plateau path after each softening period. This explains the homogenized elastic behavior and the rapid collapse of the cells as aforementioned in the previous sections, as elastic recovery ability was dominated by ductile damage failure. These results are in agreement with low strain-rate experimental studies comparing the specific energy absorption of the three most common TPMS topologies, where Diamond exhibits the highest value, followed by Gyroid and Primitive [9,18]. Experimental studies on other metal-based lattice structures have also shown the superiority of TPMS structures over strut-based structures in terms of energy absorption [9].

**Figure 10:**
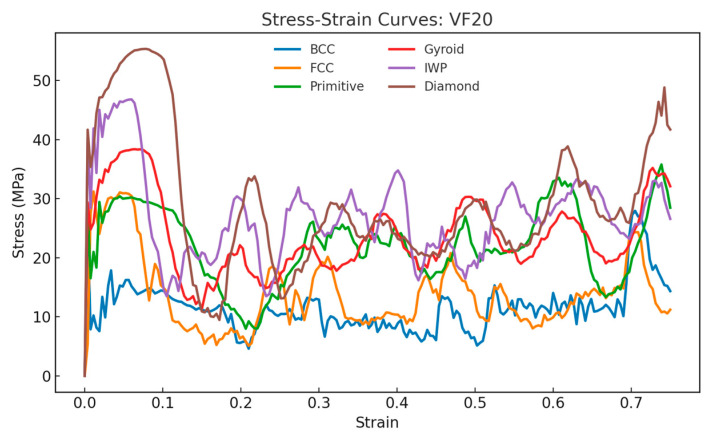
Stress–strain curves and energy absorption comparison between lattices under compressive loading for relative density of 20%.

**Figure 11:**
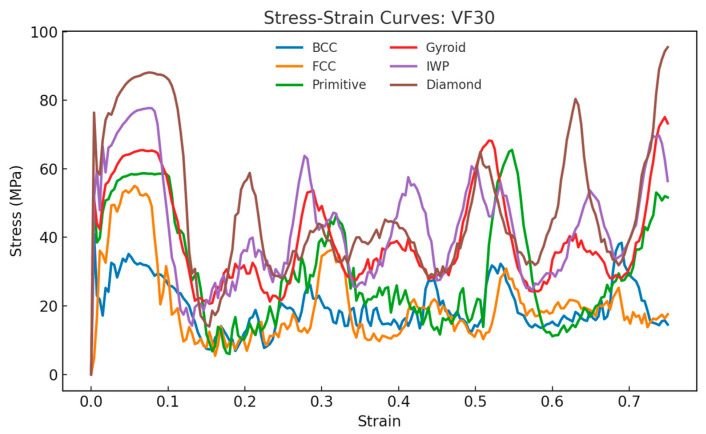
Stress–strain curves and energy absorption comparison between lattices under compressive loading for relative density of 30%.

**Figure 12:**
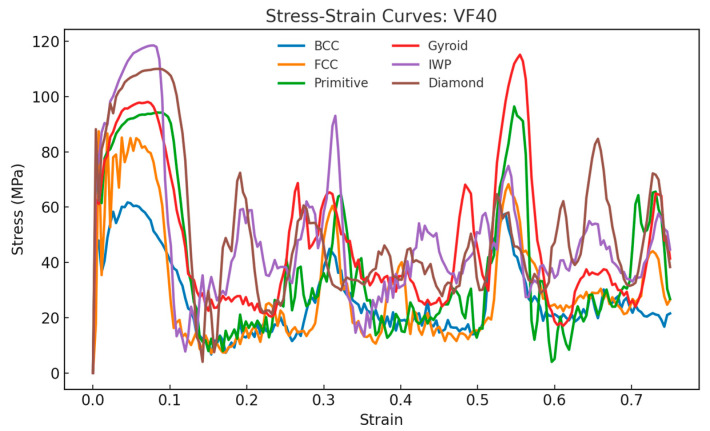
Stress–strain curves and energy absorption comparison between lattices under compressive loading for relative density of 40%.

Figure 10, Figure 11 and Figure 12 show the compressive stress–strain curves for all lattice types at 20%, 30%, and 40% relative density, respectively. The characteristic oscillatory behavior is evident across all TPMS- and strut-based lattices, with multiple load drops corresponding to localized cell wall collapse followed by stress recovery. This oscillatory behavior is consistent with experimental studies on the compressive response of AlSi10Mg lattices [37,38]. Diamond exhibited the highest peak stress and most pronounced recovery, suggesting a superior resistance to localized fracture.

### 3.5. Ductile Failure and Damage Evolution Analysis

**Figure 13:**
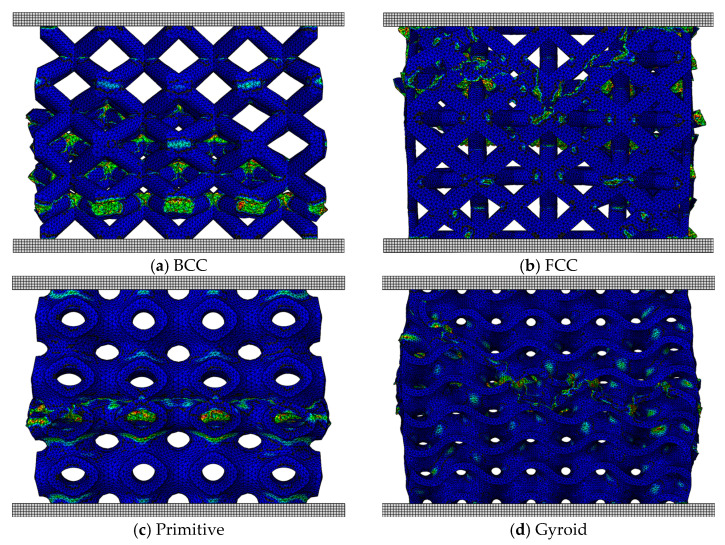

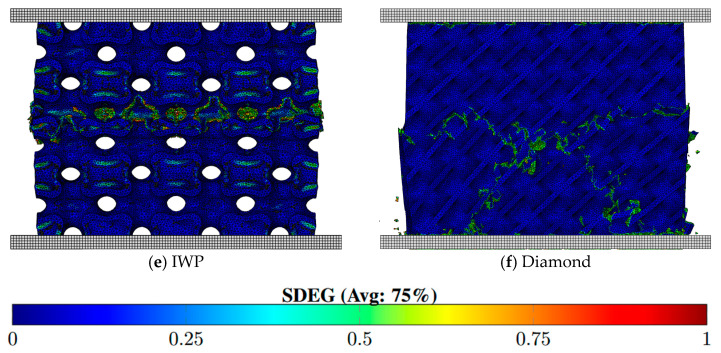
SDEG in lattices with relative density ρ=30% at strain ε=0.2.

To better understand the failure mechanisms and to locate the fracture zones, the stiffness degradation (SDEG) is visualized. At damage initiation, the value of SDEG is equal to 0. As the material undergoes softening and then fracture, stiffness degradation increases to 1. Mapping the SDEG provides insights into the evolution of local stiffness loss and fracture zones. Figure 13 shows the distribution of SDEG at a global strain of 0.2 for each lattice type at 30% relative density.

The strut-based lattices, BCC and FCC, exhibit distinct failure modes despite their geometric similarities. The BCC lattice fails over a large sub-volume, forming a shear band in three dimensions, whereas FCC fails in a V-shape. In contrast, TPMS lattices spread out the compressive load more evenly over a larger volume, thus delaying the onset of local failures. Schwarz Primitive and IWP visually fail in a layer-by-layer basis, while Gyroid and Schwarz Diamond fail globally, with “X”-shaped fracture bands. This fracture pattern was particularly evident in the Diamond structure, especially at lower relative densities.

The observed failure modes and fracture mechanisms are consistent with experimental studies of metal-based lattices [39,40], where TPMS lattices demonstrated improved damage tolerance compared to strut-based structures. The continuous curvature of TPMS promotes a more homogeneous stress distribution, thereby avoiding early localization and collapse. In particular, the Diamond topology’s X-shaped failure bands confirm its ability to dissipate energy through distributed damage, supporting its superior performance in both volumetric and specific energy absorption.

## 4. Conclusions

This study presents a numerical investigation of TPMS-based lattice structures fabricated using PBF-LB/M of AlSi10Mg, comparing their mechanical properties and energy absorption characteristics to traditional strut-based lattices. The goal was to evaluate not only the effective elastic properties (Young’s modulus, shear modulus, Poisson’s ratio) but also the energy absorption characteristics under compressive loading as per standard definitions, relevant for high strength-to-weight-ratio and crashworthiness applications.

Finite Element simulations were conducted in ABAQUS. A mesh convergence study was performed to determine the element size, so that further refinement would not change the output much and keep the computational cost reasonable. This was accompanied by a study of the Representative Volume Element (RVE) size, and it was found that a 4 × 4 × 4 bulk configuration was enough to capture the effective behavior without incurring extra computational overhead. For small deformation elastic behavior, periodic boundary conditions were applied on single unit cells using quadratic tetrahedral elements (C3D10). The mechanical behavior was then homogenized according to the Hill–Mandel macro-homogeneity conditions to ensure a rigorous link between microscale deformation and macroscale response.

To characterize the plastic behavior and failure mechanisms, the Johnson–Cook plasticity model was used along with ductile damage initiation and evolution laws. A simplified ductile damage evolution law was considered using a constant damage initiation threshold. The yield stress, hardening coefficient, strain-rate sensitivity, and fracture energy were taken from literature and manufacturer datasheets. The results showed clear trends. TPMS structures outperformed strut-based designs in almost all cases. Among the TPMS geometries, Schwarz Diamond and IWP showed high energy absorption values across different relative densities. Strut-based lattices like BCC and FCC had extended plateau regions after yielding but lacked the elastic recovery and homogenized stress distribution seen in TPMS designs. This confirms the theoretical advantages of TPMS lattices, which derive their mechanical robustness from a combination of stretching- and bending-dominated zones distributed across the unit cell.

In summary, the study provides guidelines for designing and simulating AM lattice structures for mechanical performance. It highlights the need for proper boundary conditions, mesh resolution, and unit-cell size for homogenization. Most importantly, it shows the mechanical superiority of TPMS lattices, especially for energy absorption applications where structural integrity and mass efficiency matter. These results can be used as a reference for future experimental and industrial work on optimized lattice configurations for aerospace, automotive, biomedical, and high-precision robotics applications where mechanical response and material efficiency are key.

While the finite element simulations presented in this study provide valuable insights into the mechanical behavior of AlSi10Mg TPMS structures, it is acknowledged that the results have not yet been experimentally validated. The mechanical properties obtained from the simulations, therefore, represent qualitative indicative trends rather than quantitative predictions. Quantitative comparisons with experimental results remain a critical next step. Achieving this requires the calibration of the material model using tensile, notched tensile, and compression specimens, so the simulation results can be directly compared to the experimental data. While this step is beyond the scope of the current study, it will be the focus of future work to validate the numerical findings and analyze any discrepancies.

## Figures and Tables

**Table 1:** Elastoplastic material constants for AlSi10Mg.

	General	Elastic Properties		Johnson–Cook Plasticity			
Property	ρ (kg/m^3^)	E (GPa)	v	A (MPa)	B (MPa)	n (-)	C (-)
Value	2700	71	0.33	369	684	0.73	0.0083

**Table 2:** Ductile damage constants for AlSi10Mg.

	Damage Initiation and Evolution		
Property	$\varepsilon_{f}$	η	$G_{c}$ (N/mm)
Value	0.11	−0.33	10

## Data Availability

The original contributions presented in the study are included in the article, further inquiries can be directed to the corresponding author.

## Abbreviations

The following abbreviations are used in this manuscript:

AM	Additive Manufacturing
CAD	Computer-Aided Design
FEA	Finite Element Analysis
MPE	Mean Percentage Error
PBF-LB/M	Laser Powder Bed Fusion
RUC	Repeated Unit Cell
RVE	Representative Volume Element
SDEG	Stiffness Degradation
SEA	Specific Energy Absorption
STL	Stereolithography
TPMS	Triply Periodic Minimal Surface
